# A Computational Framework for the Automated Construction of Glycosylation Reaction Networks

**DOI:** 10.1371/journal.pone.0100939

**Published:** 2014-06-30

**Authors:** Gang Liu, Sriram Neelamegham

**Affiliations:** Department of Chemical and Biological Engineering, and The NY State Center for Excellence in Bioinformatics and Life Sciences, State University of New York, Buffalo, New York, United States of America; Texas A&M University, United States of America

## Abstract

Glycosylation is among the most common and complex post-translational modifications identified to date. It proceeds through the catalytic action of multiple enzyme families that include the glycosyltransferases that add monosaccharides to growing glycans, and glycosidases which remove sugar residues to trim glycans. The expression level and specificity of these enzymes, in part, regulate the glycan distribution or glycome of specific cell/tissue systems. Currently, there is no systematic method to describe the enzymes and cellular reaction networks that catalyze glycosylation. To address this limitation, we present a streamlined machine-readable definition for the glycosylating enzymes and additional methodologies to construct and analyze glycosylation reaction networks. In this computational framework, the enzyme class is systematically designed to store detailed specificity data such as enzymatic functional group, linkage and substrate specificity. The new classes and their associated functions enable both single-reaction inference and automated full network reconstruction, when given a list of reactants and/or products along with the enzymes present in the system. In addition, graph theory is used to support functions that map the connectivity between two or more species in a network, and that generate subset models to identify rate-limiting steps regulating glycan biosynthesis. Finally, this framework allows the synthesis of biochemical reaction networks using mass spectrometry (MS) data. The features described above are illustrated using three case studies that examine: i) O-linked glycan biosynthesis during the construction of functional selectin-ligands; ii) automated N-linked glycosylation pathway construction; and iii) the handling and analysis of glycomics based MS data. Overall, the new computational framework enables automated glycosylation network model construction and analysis by integrating knowledge of glycan structure and enzyme biochemistry. All the implemented features are provided as part of the Glycosylation Network Analysis Toolbox (GNAT), an open-source, platform-independent, MATLAB based toolbox for studies of Systems Glycobiology.

## Background

Glycosylation is among the most common post-translational modifications in nature. A vast majority of cell-surface and secreted proteins in mammalian cells bear glycans [1]. These carbohydrate modifications have important functions in regulating cell recognition, signaling and adhesion processes that condition both normal mammalian development and physiology, and the progress of diseases like cancer, inflammation and thrombosis [2]. In addition, protein glycosylation regulates the half-life of therapeutic molecules in blood circulation, and thus methods to regulate the pattern of site-specific glycosylation are of considerable interest to the pharmaceutical community [3].

Unlike nucleic acids and proteins which are predominantly composed of template-driven linear polymeric structures, glycans are often composed of branched structures. Due to this complexity, a heterogeneous population of carbohydrate structures may be found at a single protein site. Thus, instead of having a uniform chemical structure, the glyans added to proteins are composed of a group of chemically related entities or ‘glycoforms’ [2]. The precise distribution of these glycoforms depends on several factors including, but not limited to: i) The location, sequential presentation, expression, concentration and activities of enzymes that synthesize these post-translational modifications in sub-cellular compartments. These enzymes, called ‘glycosyltransferases’, catalyze the transfer of monosaccharide residues from sugar-nucleotide donors to peptide substrates. Examples include sialyltransferases which decorate glycoconjugates with sialic acid like N-Acetylneuraminic acid (Neu5Ac) and fucosyltransferases which add fucose (Fuc). ii) The competition between different families of enzymes for common substrates. For example, the N-acetyl lactosamine structure (Galβ1,4GlcNAc, i.e., Galactoseβ1,4N-Acetylglucosamine) can act as a substrate for enzymes belonging to both the α(2,3)-sialyltransferase and α(1,3)-fucosyltransferase families. iii) The competition between different members of the same family. In this regard, more than one α(2,3)-sialyltransferase may exist in cells that can act on the N-acetyl lactosamine substrate. iv) The availability of synthases, epimerases and transporters that can regulate the local concentration and distribution of sugar-nucleotide donors. v) The expression levels of ‘glycosidases’, which release monosaccharides from glycoconjugate substrates. In addition, the concentrations of the expressed protein substrates and the glycosylating enzymes depend on the cellular growth and differentiation status.

There has been a large effort in the Glycomics field over the last decade to capture the complexity of the glycosylation process using high throughput analytical experimental methods, notably mass spectrometry [4], and also arrays that have immobilized glycans or lectins [5], [6]. Such efforts have been applied to profile the glycan structures on whole cells like the Chinese Hamster Ovary cells (CHO) [7] and also animal organ systems [8] in response to a variety of molecular perturbations. Whereas these methods help profile the overall glycome of cells and organ systems, they do not provide a detailed quantitative understanding of the complex glycosylation machinery that drives such syntheses. Systems biology approaches have been proposed to overcome this limitation by developing systems-based mathematical models [1], [9]–[15].

**Figure 1:**
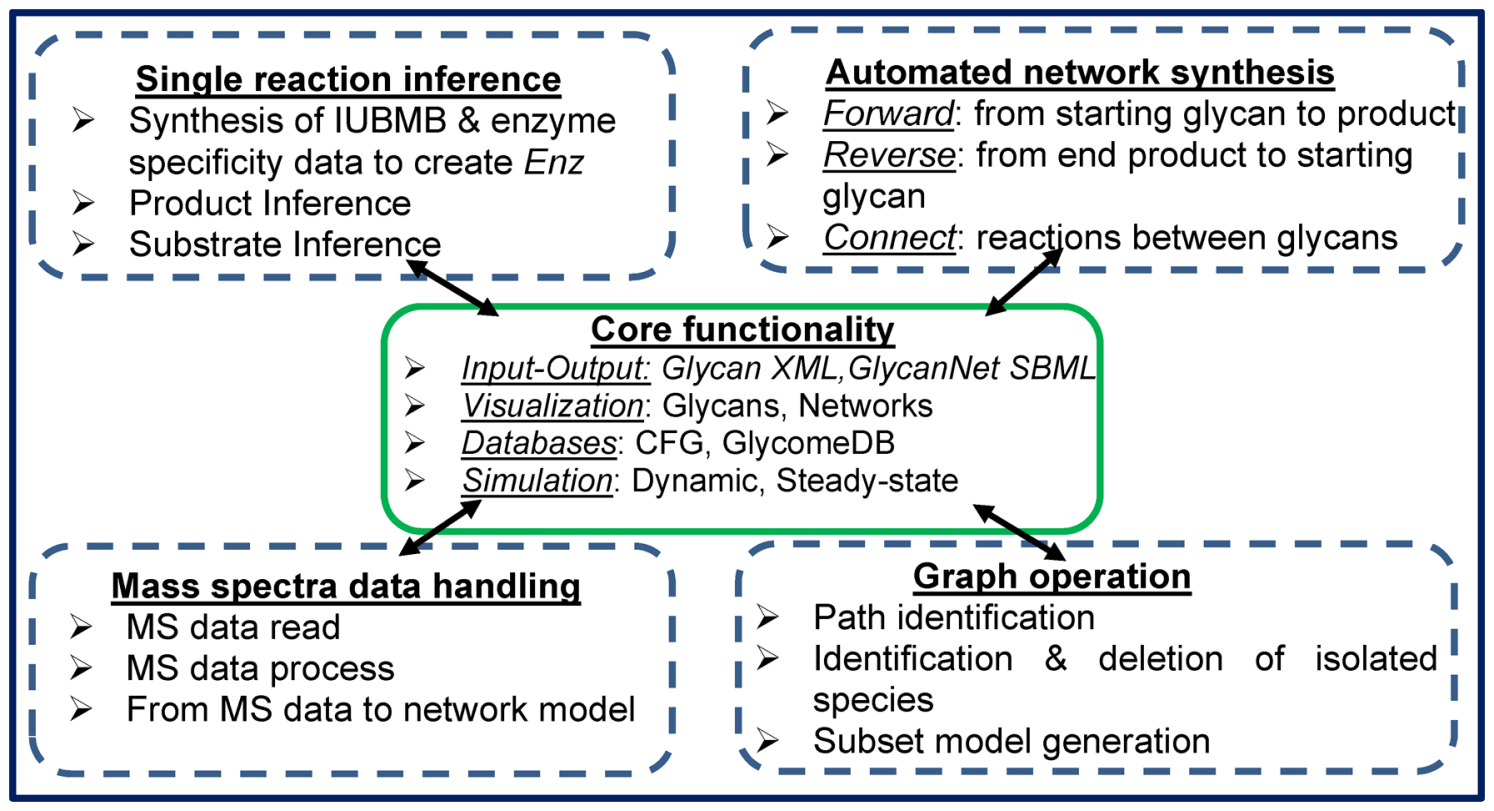
Overview of the computational framework for glycosylation network construction. Green solid center box presents the core functionality provided in GNAT. Surrounding this are four blue dashed boxes that highlight network construction features that are enabled by the definition of enzymes in machine-readable format.

The construction of glycosylation reaction networks *in silico* is an important step that can enable the quantitative analysis of biochemical experimental data. In this regard, the manual construction of such networks can be tedious and error-prone, particularly when the network size is large. Recent efforts have attempted to address this problem [10], [13]. However, the scope of these approaches is limited due to the lack of a systematic definition for the absolute, group, linkage and stereochemical specificity of glycosylating enzymes. In the current manuscript, we overcome these limitations by defining a comprehensive *Enzyme* class that integrates glycosyltransferase and glycosidase enzymatic data from the IUBMB (International Union of Biochemistry and Molecular Biology) database and substrate specificity information from a variety of sources (top-left blue box with dashed line, Figure 1). As a result, both single reaction inference and network reconstruction algorithms are now feasible (top-right blue box, Fig. 1). In one application, we demonstrate how these new facilities allow the construction of network models using mass spectrometry data (bottom-left blue box, Fig. 1). In another application, we demonstrate how the new class definitions allow subset model generation and other advanced model analysis strategies (bottom-right blue box, Fig. 1). These new facilities are integrated into GNAT (Glycosylation Network Analysis Toolbox), a comprehensive open-source toolbox for the field of Systems Glycobiology [14].

## Computational Design and Methods

The current manuscript describes a systematic method to define the properties of glycosyltransferases and glycosidase enzymes using object-oriented programing concepts. It streamlines four major processes (blue boxes in Figure 1): i) Automated construction of single glycosylation reactions using enzyme definitions and biochemical experimental data; ii) Extension from single reactions to automated construction of entire glycosylation reaction networks; iii) Graph operations to examine network flow and connectivity; and iv) Incorporation of glycan experimental data, specifically mass spectrometry based glycomics data, into the simulation environment.

The strategy to describe enzymes in machine-readable format along with the four methodological advancements is described in detail below. Associated classes and methods included in the GNAT software package are also described. Note that the classes and variables appear in *plain italicized* fonts and functions appear in ***bold italicized*** fonts in this manuscript. An exhaustive manual provided with the software includes the detailed theory, user guide, demos and examples.

### Machine-readable definition of enzymes

**Figure 2:**
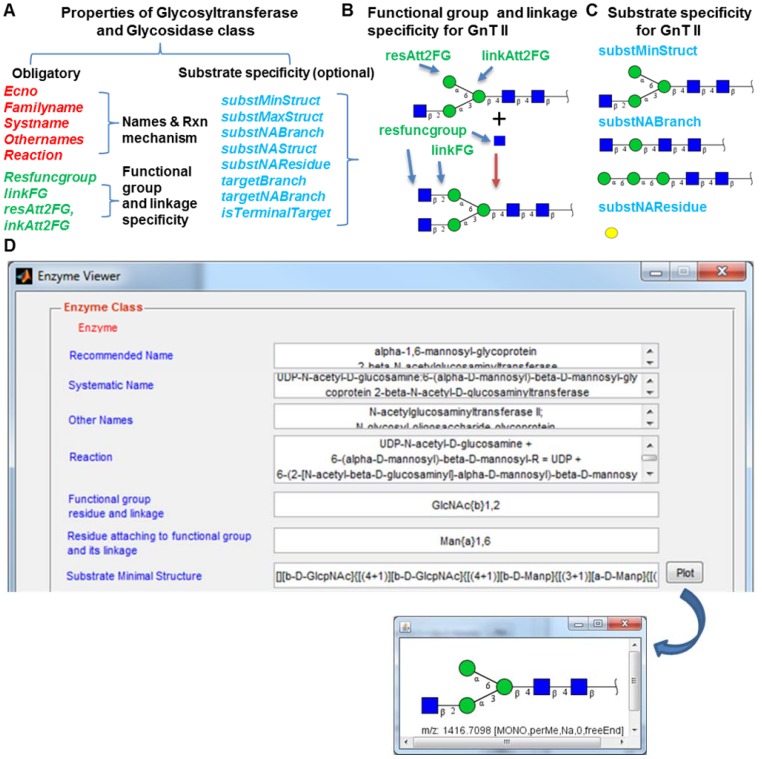
The *Enz* class. Glycosyltransferases (*GTEnz*) and glycosidases (*GHEnz*) are subclasses of the parent *Enz* class. **A**. They contain fields that are present in *Enz* (red) and also additional obligatory fields that describe the glycosylation reaction (green). *resAtt2FG* and *linkAtt2FG* are optional for *GHEnz*. In addition, the sub-class contains properties that describe additional optional details regarding enzyme specificity (blue). **B**. Description of the *resfuncgroup, linkFG, resAtt2FG* and *linkAtt2FG* using β1,2-N-acetylglucosaminyltransferase (GnT II) as an example. **C**. Examples of optional fields that constrain the substrate specificity of GnTII. **D**. Screenshot of a portion of the Enzyme Viewer window showing GnT II properties. Substrate specificity data are presented in LINUCS format. Clicking the ‘Plot’ button on right opens a window to display the glycan structure. Color code used to describe glycans follow the Consortium of Functional Glycomics nomenclature (www.functionalglycomics.org).

Data from IUBMB and other literature sources are used to describe the enzymes in machine-readable format (Fig. 2). In GNAT, the generic enzyme is described using the enzyme or *Enz* class. This class contains the Enzyme Commission Number (ECNO), enzyme names (systematic, recommended, and alternative name), and reaction description (red text, Fig. 2A). Many of these properties are automatically populated by querying the IUBMB enzyme database. The results can be visualized in a new ‘Enzyme Viewer’ GUI that is included in GNAT (Fig. 2D). This viewer displays fields for *Enz* and also children classes described below.

The transferase (*TfEnz*) and hydrolase (*HlEnz*) are subclasses of *Enz*. They inherit properties from *Enz*. Additionally, in order to describe the reaction type catalyzed by these enzymes, as shown below, these subclasses include fields that store *donor*, *acceptor*, *donorprod* and *acceptorprod* in the case of *TfEnz* and *prod_h* and *prod_oh* in the case of *HlEnz*.

Transferase (*TfEnz*) reaction: 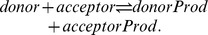


Hydrolase (*HlEnz*) reaction: 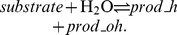


*GTEnz* and *GHEnz* are enzyme classes that specifically describe the glycosyltransferases and glycosidases respectively. These classes inherit properties defined in *TfEnz* and *HlEnz* respectively. In addition, properties are provided in these classes in order to describe the detailed enzyme specificity (green and blue text, Fig. 2A). Among these, *resfuncgroup* and *linkFG* describe the glycosidic bond and its linkage that is either formed by the *GTEnz* or that is broken by *GHEnz* (Fig. 2B). This figure presents a schematic where GlcNAc (N-acetylglucosamine) is added to an N-linked glycan using the β1,2-N-acetylglucosaminyltransferase, GnT II. Here, *resAtt2FG* and *linkAtt2FG* define the glycosidic bond adjacent to this linkage. The other properties of *GTEnz* and *GHEnz*, listed in Fig. 2A, define additional structural elements of the substrate or product that constrain the feasibility of the reaction. For example, *substNAResidue* describes the specified residues in the substrate structure that prevent enzyme activity, and *targetBranch* describes the substrate characteristics of the target branch where the enzyme acts. These structures are displayed in LINUCS (Linear Notation for Unique description of Carbohydrate Sequences) format in Fig. 2D [16], or they can be graphically viewed by clicking the plot button of the Enzyme Viewer. This visualization component, in part, uses the GlycanBuilder library [17]. Additional properties of the *GTEnz* and *GHEnz* classes can be viewed in the source code or GNAT user manual. Detailed documentation about these classes can be also accessed using the *help* or *doc* commands provided in MATLAB. Note that enzyme rate constants required for kinetic modeling have to be user-defined based on experiments or prior literature. These are not automatically populated by GNAT.

### From enzyme classes to single reaction inference

Definition of the glycosyltransferase (*GTEnz*) and glycosidase (*GHEnz*) class allows automated single-reaction ‘product/substrate inference’ (top-left, Fig. 1). In the case of ‘product inference’, the product of glycosyltransferase/glycosidase reactions can be automatically determined if a substrate and enzyme are specified. Similarly, in the case of ‘substrate inference’, the reverse inference can be performed where the starting reactant is determined given a product and enzyme. In the GNAT package, such single-step reaction inference is performed by following a three step algorithm that includes: 1) Verification that a given enzyme can act on a candidate glycan-substrate; 2) Identification of residues that can be enzymatically modified; and 3) Creation of reaction product based on rules described in *GTEnz/GHEnz*. The details regarding the algorithm and its usage syntax are provided in the GNAT manual.

### From single reaction inference to full glycosylation reaction network construction

**Figure 3:**
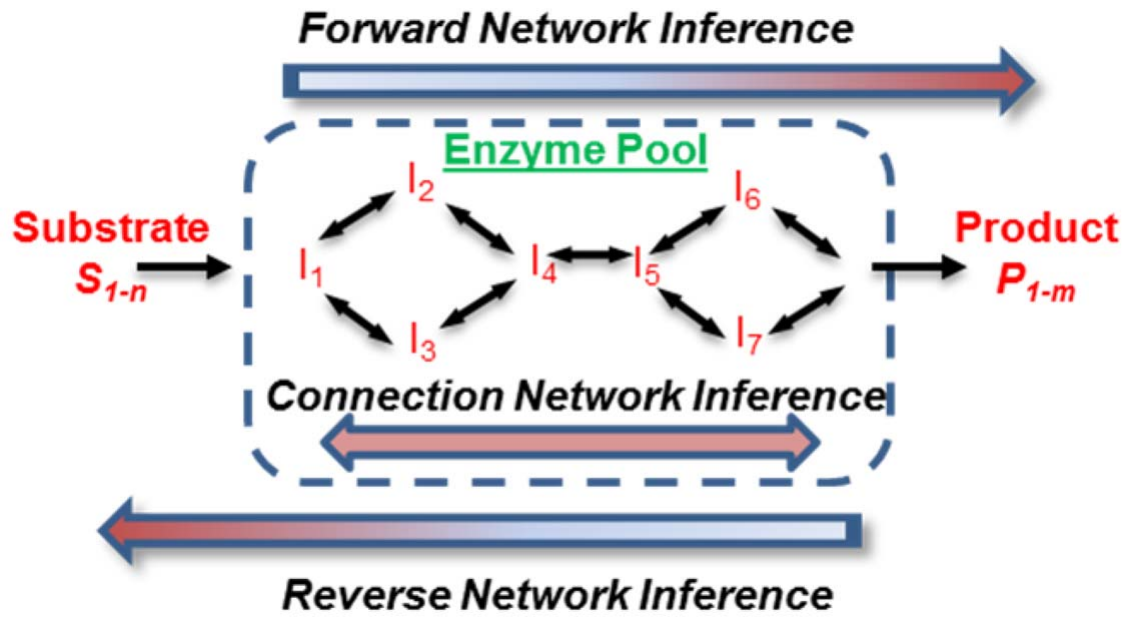
Automated network inference. Cell (depicted as dashed blue box) contains a pool of enzymes and related enzyme properties. It is possible to infer *in silico*: i) the intermediate reactions and products if a set of substrates is provided (‘forward network inference’); ii) the starting substrate and intermediate reactions if a set of products is provided (‘reverse network inference’); or iii) the intermediates formed given a set of starting and ending glycans (‘connection network inference’).

The newly-designed glycosyltransferase and glycosidase classes, along with methods for single reaction construction in the previous section have been extended to enable automated glycosylation pathway construction (Top-right Figure 1, Figure 3). Three types of network inference methods are implemented: i) ‘Forward network inference’: Here, intermediate reactions and products are inferred when a set of starting substrates and enzymes are specified; ii) ‘Reverse network inference’: Here, the starting material and reactions are inferred given a set of products and enzymes; and iii) ‘Connection network inference’: Intermediate reactions that connect two or more glycans are inferred here, using enzyme-substrate specificity data provided in the *GTEnz* and *GHEnz* classes.

For network construction, the algorithm for substrate and product reaction inference is similar. In the case of ‘forward network inference’, starting with one of the starting glycans, single-step ‘product inference’ is applied in a sequential manner. This is repeated until no additional new product(s) are formed in a given step for the set of enzymes specified in the system. All newly generated molecules and reactions generated in this manner are incorporated into the reaction *Pathway* object. The above process is then repeated for each of the starting glycans. ‘Reverse network inference’ is similar, only it starts with the network products and applies ‘substrate inference’ to determine the starting glycans.

‘Connection network inference’ joins two or more input glycans using enzyme-substrate specificity data defined in the *GTEnz* and *GHEnz* classes. The algorithm initially chooses a pair of glycans. For this pair, based on the enzymes present in the system, the program designates one of the molecule to be the ‘initial substrate/starting material’ while the other is the ‘final product’. For the initial substrate-final product pair, ‘substrate inference’ is applied in a step-wise manner in order to determine all possible reaction pathways that link the final product to the initial substrate. For *m* glycans provided as input, the above steps are repeated *m*×(*m*-1)/2 times. Finally, all the reactions and species are consolidated into the *Pathway* object by removing duplicate reactions and species.

Pathways generated by the above algorithms are exported in System Biology Markup Language (SBML) format files [18]. These XML based files, list reactions and species in the pathway. Here, glycan structure information is included in the *annotation* field of the *Species* element as shown below:

<species>

…

<annotation>

  <glycoct xmlns = “http://www.eurocarbdb.org/recommendations/encoding”>

   <sugar version = “1.0”>

    <residues>

    …

    </residues>

   <linkages>

   …

   </linkages>

  </sugar>

 </glycoct>

</annotation>

…

</species>

The constructed pathway can be graphically visualized using the glycosylation network visualizer (GNV), a visualization component of GNAT. GNV is a Java-based library that uses JGraph to display network structures (http://www.jgraph.com/) and GlycanBuilder [17] to display individual glycans.

### Graph operations for network structure analysis

Due to the branched nature of glycans and the broad specificity of glycosidases/glycosyltransferases, a variety of reaction routes can link a single starting glycan to a given product. Thus, upon constructing an overall glycosylation reaction network using a group of experimentally measured glycans, the inferred network may be unwieldy with hundreds or thousands of reactions and products [13]. Not all these reaction routes may be of equal biological significance. For this reason, it is attractive to systematically construct and analyze a subset of the overall reaction pathway in order to identify biologically significant elements. Using the streamlined *GTEnz* and *GHEnz* classes, graph theory methods are now implemented for such analysis (bottom-right, Figure 1).

In GNAT, the *Pathway* class has been designed to mimic graph data structures in order to enable subset pathway analysis. In this data structure, the glycan species represent the nodes of the graph and the edges correspond to the biochemical reactions. A number of high-level graph operation algorithms are implemented in GNAT, and two of these are described below:

i. The ‘***pathfinding***’ function determines all pathways that connect two species in a reaction network. This algorithm applies the depth-first search method to find the reaction path between the two inputs. Here, the first child node of the ‘starting species’ is identified and checks are performed to determine if the child is the ‘target glycan/species’. If not, the algorithm continues to examine the children of this first child, and this proceeds stepwise deeper and deeper until either the target node is identified or a node lacking children is reached. The latter being the case, the search returns back to the most preceding species and it visits additional routes. The process proceeds recursively, until all possible connecting links from the ‘starting species’ have been examined. All searches that identify a link between the ‘starting’ and ‘target’ species are stored.

ii. A set of ‘subset generation’ functions enable the dynamic synthesis of reaction networks. These functions construct small reaction networks by extracting portions of the larger ‘master’ reaction network. In the first method (***subsetgenbynumdel***), the systematic removal of user-specified number of species results in a set of smaller reaction networks where the specified number of species has been randomly deleted. In the second and third methods, user-specified species are either deleted using the ***subnetgenbyspecdel*** function or retained using the ***subnetgenbyspeckeep*** function. These methods represent alternate strategies to generate subset reaction networks.

### Generation of reaction networks using MS-based glycomics data

The implementation of the *GTEnz* and *GHEnz* classes along with the network inference and analysis algorithms above suggest that it is possible to construct glycosylation reaction networks *in silico* if glycan structure and enzyme specificity data are available. A number of experimental protocols may be applied to obtain such information. Mass spectrometry based glycomics experiments that profile per-methylated glycans released from either individual proteins [19], cells [7] or tissue [8] represent one such strategy.

Two basic MS functions are implemented in GNAT in order to read raw glycomics experimental data and preprocess them in order to identify MS peaks corresponding to the glycans. To this end, the raw data processing function enables loading of raw mass/charge (*m*/*z*) and intensity data from.msd format files into the computational environment. The peak selection function then converts the raw *m*/*z* vs. intensity data into a list of peaks and their corresponding half height width column data using a four-step method. These steps include background adjustment, data normalization, noise removal, and peak finding. Peaks identified in this manner can be related to specific glycans using algorithms described elsewhere [7], [8], [19]. When enzyme data for specific cell type are gathered from literature, it is feasible to construct a protein/cell/tissue-type specific glycosylation network using glycan structures annotated from the processed MS data.

## Results

The definition of the *GTEnz* and *GHEnz* classes within the GNAT environment enables the systematic creation of enzyme databases that can collate enzyme specificity data available in literature [20]. For the purpose of the current study, we created a small database with 2 glycosidases and 12 glycosyltransferases that participate in O- and N-linked glycosylation reactions. This database was applied to test the automated network generation computer algorithms described in the previous section. Three case studies are presented below to illustrate this effort. These describe: 1) O-linked glycosylation reaction network construction and subset modeling; 2) complex N-linked pathway generation using the ‘forward network inference’ method; and 3) automated network construction from MS data.

### Case Study 1: O-linked glycosylation networks

O-linked glycans attached to the leukocyte cell surface molecule P-selectin glycoprotein ligand-1 (PSGL-1) bind adhesion molecules belonging to the selectin family [21]. This molecular recognition regulates leukocyte-endothelial cell adhesion during inflammation. Sialyl Lewis-X (sLe^X^) (Neu5Acα2,3Galβ1,4(Fucα1,3)GlcNAc) attached to a core-2 structure on PSGL-1 is a key glycan that initiates this cellular attachment. Glycosylation reaction network analysis can be performed to study the biosynthetic routes regulating the formation of this glycan in order to define key rate-limiting steps [10].

**Figure 4:**
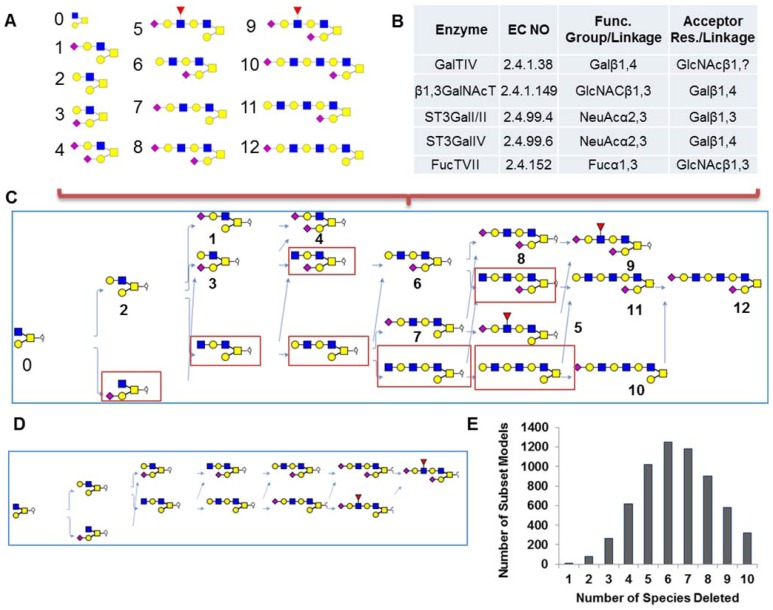
O-linked glycosylation network construction and analysis. **A.** List of glycans determined to be present in the cell based on biochemical experiments. **B**. Enzyme pool in cell. **C**. ‘Connection reaction inference’ was used to generate the ‘master pathway’ that contains all possible reactions and connections in the system. Figure generated using the ***glycanPathViewer*** function of GNAT. **D**. Path finding function determines the reaction pathway between the core-2 trisaccharide (glycan 0) and glycans containing the sLe^X^ epitope (glycans 5 and 9). **E**. Histogram plot quantifying the number of subset pathways generated when 1 to 10 O-glycans were deleted from the master pathway in panel C.

Biochemical studies have identified twelve possible O-glycans on PSGL-1 ([22], labeled 1-12 in Fig. 4A). Among these, glycans 5 and 9 bear the sialyl Lewis-X structure. In order to determine the potential pathways that construct these glycans, a reaction space containing five different enzyme activities was considered (Fig. 4B). These enzymes include the β1,4-galactosyltransferase GalT-IV, N-acetyl glucosaminyltransferase β1,3GlcNAc-T, α2,3-sialyltransferases ST3Gal-I/II and ST3Gal-IV, and α1,3-fucosyltransferase FT-VII. Fig. 4B defines the enzyme specificity rules for each of these enzymes. Using the 13 glycans (the core-2 trisaccharide ‘starting glycan’ and 12 experimentally identified structures) and five enzyme definitions, an O-linked glycosylation pathway leading to formation of the PSGL-1 glycans was constructed using the ‘connection network inference’ function (***inferGlyConnPath*** in GNAT) (Fig. 4C). The pathway generated using GNAT has 20 species and 28 reactions. This includes the starting core-2 trisaccharide, 12 experimentally determined glycans, and 7 ‘intermediate’ structures generated *in silico* (shown in red box) that are predicted to exist based on enzyme specificity rules.

Fig. 4D depicts a portion of the master pathway that illustrates the reaction route leading from the core-2 trisaccharide to the sLe^X^ bearing glycan number-9. This smaller pathway, which is a subset of the larger pathway shown in Fig. 4C, was created using the ***pathfinding*** function of GNAT.

It is possible that all reactions identified in a given network may not have a significant contribution to the composition of the output glycans or the ‘system output’. One method to identify the important contributors is to eliminate either one single or a group of reactions in the network, and then quantifying the relative impact of such perturbations on system output. Indeed this is the spirit of gene knockout wet-lab experiments. The subset modeling functions of GNAT allows such experimentation *in silico*. In this regard, GNAT provides a range of functions to generate subset pathways by either eliminating a fixed number of species, retaining or deleting specified species. During this process, any reactant(s) that is not connected to the remaining pathway are determined using the ***detectIsolatedSpecies*** method of GNAT and they are deleted using ***removeIsolatedSpecies***. In the O-linked reaction pathway, deleting between 1–10 species using the subset network generation function generated a set of subset pathways. These subset pathways all start with the core-2 trisaccahride glycan 0 and contain at least one of the sLe^X^ species 5 or 9. Further, all ‘intermediate’ structures generated *in silico* (red box, Fig. 4C) are both the reactant and the product of at least one reaction. As seen in Fig. 4E, the number of subset pathways increases with the number of species deleted with a maximum when 6 species are deleted. Rate limiting steps that control system output can be identified by analysis of subset pathway networks.

### Case Study 2: Theoretical prediction of N-linked glycosylation pathways

**Figure 5:**
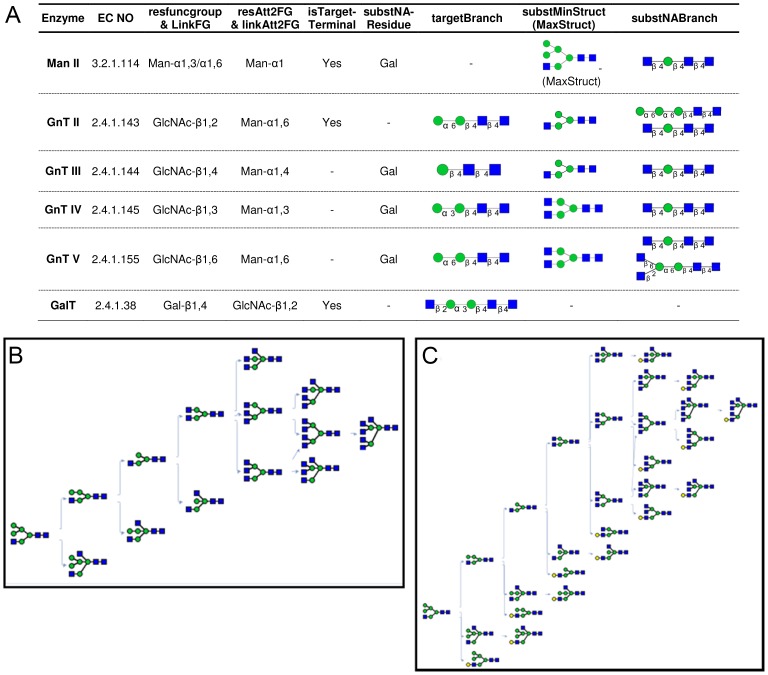
N-linked glycosylation pathway reconstruction. **A.** Enzyme rules are defined for a pool of six enzymes: GnT II, III, IV, V, ManII and GalT. ‘-’ indicates blank fields in the *GHEnz*/*GTEnz* class definitions. **B**. N-linked glycosylation pathways generated using the ‘forward network inference’ function, when GlcNAcMan_5_GlcNAc_2_ was the starting substrate and five enzymes defined in panel A (GnT II-V and Man II) were used. **C**. Network generated using all six enzymes in panel A.

The biosynthesis of N-linked glycans is initiated by the *en bloc* transfer of the dolichol-linked precursor, Glc3Man9GlcNAc2 (Glucose3-Mannose9-GlcNAc2) in metazoans, to asparagine residues on nascent proteins in the endoplasmic reticulum (ER) [2]. It is a branched glycan that includes a Man3GlcNAc2 core sequence that is common to all N-glycans that are later formed. Following this transfer, glucose and mannose residues are trimmed from the precursor to form oligomannose structures like Man9GlcNAc2 (M9) and Man8GlcNAc2 (M8) in the ER. These are transported to the Golgi for additional biochemical processing. The reactions in the Golgi involve the additional trimming of mannose residues and the addition of GlcNAc, galactose, fucose and sialic acids to the glycan. N-glycan branching is an important biosynthetic step during this elaboration since it results in products with a varying number of ‘antennae’. Such branching is mediated by a family of N-acetylglucosaminyltransferases or GlcNAcTs which exhibit highly-specific substrate specificity. Five of the common members of this enzyme family include GnT I (α1,3-mannosyl-β1,2GlcNAcT I), GnT II (α1,6-mannosyl-β1,2GlcNAcT II), GnT III (β1,4-mannosyl-β1,4GlcNAcT), GnT IV (α1,3-mannosyl-β1,4GlcNAcT) and GnT V (α1,6-mannosyl-β1,6-GlcNAcT) (Fig. 5A). These are encoded by genes that are called *MGAT*1*-5*. Branching by these enzymes and additional terminal modifications result in the formation of a variety of carbohydrate structures. The end products of such synthesis include three types of structures called oligomannose, hybrid and complex N-glycans, each containing the common Man3GlcNAc2 core structure. In this regard, oligomannose glycans have only mannose residues attached to the core. Some branches of hybrid N-glycans are mannose rich while others are extended by the addition of GlcNAc. Complex glycans have GlcNAc attached to all antennae.

Figure 5 presents an example of N-linked glycosylation where the starting glycan is GlcNAcMan5GlcNAc2 (M5Gn). ‘Forward network inference’ (***inferGlyForwPath***) is used here to construct the reaction network. Fig. 5A describes a system that includes five enzymes, the glycosidases Man II and the glycosyltransferases GnT II to GnT V. The detailed substrate specificity rules for these enzymes are defined in Fig. 5A based on work by others [13]. As seen here, the *targetBranch* property of these enzymes is often populated in the enzyme class, since it helps define the exquisite substrate specificity of the glycosyltransferases. In addition, the linkage specificity of Man II is described such that it can release mannose residues from both α1,3 and α1,6 linked glycans (Fig. 5A). In many reactions, the bisecting GlcNAcT (GnT III) acts on the central core mannose residue to create a β1,4 linked GlcNAc. This action inhibits both glycan trimming by α-mannosidase II and also prevents the action of other GlcNAcTs, GnT II, GnT IV and GnT V. Thus, this substrate specificity data is included in the *GHEnz* definition by providing a *substNABranch* for Man II (Fig. 5A). Also, the activities of GnT II, GnT IV and GnT V are constrained by including GlcNAcβ1,4Manβ1,4GlcNacβ1,4GlcNAc in the *substNABranch* field.

When five enzymes are provided in the reaction network, a system with 14 species and 14 reactions is formulated (Fig. 5B). The inclusion of another enzyme (GalT) results in the automatic construction of a larger reaction network with 28 species and 28 reactions, similar to the work of Umana *et al*. [15] who manually constructed this network (Fig. 5C). Here, the activity of the GalT is constrained to the terminal mannose on the lower antennae of the N-glycan using the *targetBranch* property in Fig. 5A. Further, GnT activity on glycans is prevented after galactose addition since this monosaccharide is listed under *substNAResidue* (Fig. 5A). Overall, the example of N-glycosylation illustrates how the fields of the *GHEnz* and *GTEnz* classes can be defined in order to define subtle differences in the substrate specificity of a family of related enzyme.

### Case study 3: Construction of glycosylation pathway from MS based glycomics data

**Figure 6:**
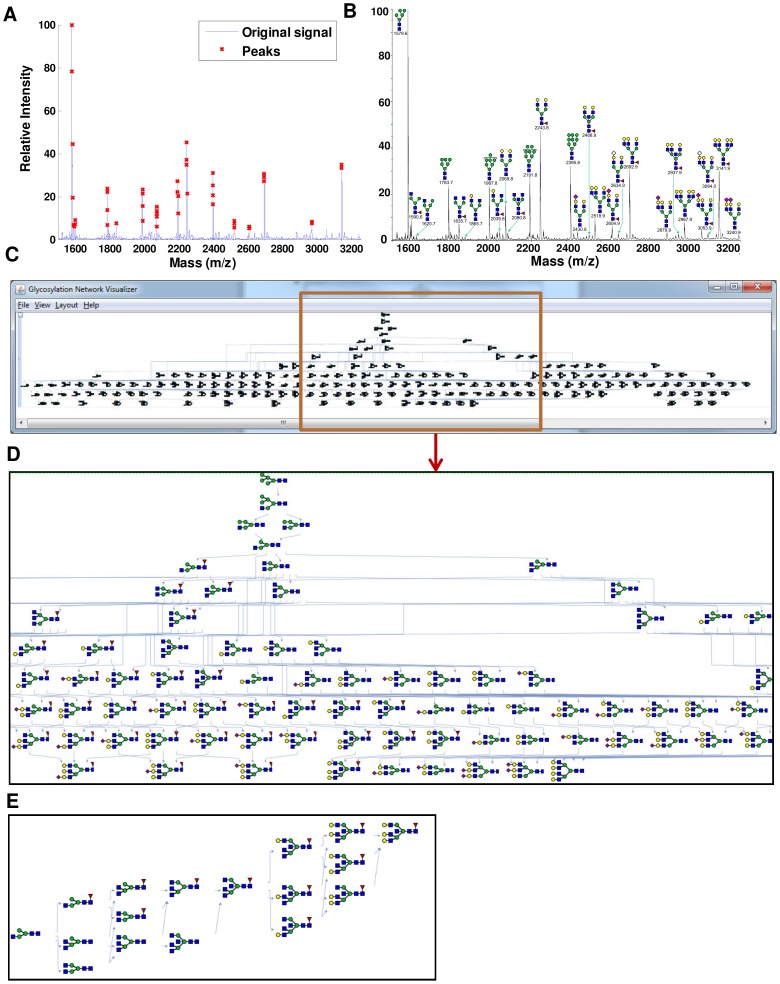
Reaction network generated from glycomics experimental data. **A.** The ***readMS*** and ***msprocess*** functions of GNAT were used to smooth raw experimental data. **B**. Glycans annotated in the MS spectra available from the Consortium of Functional Glycomics web site. **C**. ‘Connection network inference’ applied for network construction using 43 annotated input glycans and 9 enzymes (GnT I-V, ManII, FucT, SiaT, and GalT). Constructed network has 360 reactions and 188 species. **D**. Expanded view of a portion of the network. **E**. ***pathfinding*** function used to connect the starting GlcNAcMan3GlcNAc2 structure and the tri-bisecting antennary glycan with m/z  = 2937.9.

The class definitions, reaction network construction tools and model analysis methodologies of GNAT are geared towards handing mass spectrometry based glycomics data sets. This is illustrated in Figure 6 using MALDI-TOF (matrix assisted laser desorption ionization-time of flight) mass spectrometry data that profile the N-glycans of wild type CHO (Chinese hamster ovary) cells [7]. The original raw mass spectrometry data downloaded from the CFG database has N-glycans in the m/z range from 1500 to 3250 when CHO cells were grown in monolayer. This was read using the ***readMS*** function of GNAT and the data were processed using the ***msprocess*** function (Fig. 6A). The peaks corresponding to the MS data can be attributed to 20 distinct glycan compositions, with the possibility that more than one isomeric glycan may contribute at a given *m*/*z* value (Fig. 6B). Due to this uncertainty, the number of possible glycans in the system is expanded to 43.

Considering the presence of 9 enzymes, GnT I to GnTV, Man II, SiaT, FucT and GalT, ‘connection network inference’ (***inferGlyConnPath*** *function*) was applied to construct a relatively large N-linked glycosylation pathway with 360 reactions and 188 species (Fig. 6C). The starting glycan for this network was Man5GlcNAc2. Fig 6D presents an expanded view of a portion of this network. Fig. 6E uses ***pathfinding*** function to isolate the network connecting the GlcNAcMan3GlcNAc2 glycan to the tri-bisecting antennary glycan (*m*/*z* = 2937.9).

## Discussion

We present a computational framework that can automate the construction of glycosylation reaction networks using streamlined enzyme definitions. Specifically, the new methods implement machine-readable definitions for glycosyltransferases (*GTEnz*) and glycosidases (*GHEnz*). Using this and related algorithms, it is possible to construct glycosylation networks starting from either conventional biochemical or mass spectrometry based experimental data. Once constructed, a variety of network analysis strategies can be implemented to analyze components of the overall glycosylation network using graph theory based concepts, like the ‘subset modeling’ and ‘path finding’ algorithms.

The network construction and analysis algorithms described here are implemented in GNAT, the only toolbox currently available for the analysis of biochemical pathways related to the field of Glycobiology. In this regard, GNAT is a MATLAB-based, open-source, platform-independent computational toolbox for Systems Glycobiology [14]. It is different from other network analysis software listed at sbml.org since it is specifically geared towards the analysis of cellular glycosylation processes. It is also different from other computational programs in the Glycosciences like GlycomeAtlas, which is used to visualize glycome profiling data [23]. Finally, while a variety of programs are available to analyze glycomics based MS data (reviewed in [24]), GNAT is more focused on synthesizing such experimental results for quantitative biochemical reaction network simulation and hypothesis generation.

GNAT was originally designed with the goal of providing a strategy to integrate glycan structure information into SBML format files that describe biochemical reaction pathways. In particular, a variety of object oriented concepts were implemented to modularize the programming structure. By integrating JAVA based visualization suites, namely JGraph and GlycanBuilder, the core functions of GNAT allow high quality visualization of glycosylation reaction networks. In addition, since GNAT is implemented in the MATLAB environment, the software can be readily enhanced by writing additional.m scripts and plugs-ins.

Whereas the original GNAT software contained basic functions that are listed in the center box of Figure 1, it lacked a robust enzyme definition and associated methods (blue boxes at the periphery). This shortcoming is addressed in the current manuscript by implementing *Enz* and its sub-classes. These new definitions enable the construction of general enzyme databases that can be called by multiple.m scripts. While the databases that we have developed for the current manuscript contain 14 enzymes, it is conceivable that this can be expanded in the future to include the specificity of all 200+ enzymes that regulate glycan biosynthesis [20]. Besides glycosidases and glycosyltransferases, these databases can also be enhanced to contain data regarding various kinases, synthases and epimerases that regulate sugar-nucleotide biosynthesis, and transporters that control the flux of substrates and donors into the endoplasmic reticulum and Golgi compartments. The availability of small enzyme databases in the current work enabled automated glycosylation network construction using experimental glycan structure data from a variety of sources. These new advancements are illustrated in Results using three case studies that are related to N- and O-linked glycan biosynthesis.

The goal of the current manuscript is to provide a package that can systematically automate the construction of glycosylation pathways since the manual construction of such large networks can be error-prone. In this regard, the systematic definition of enzymes in the modeling framework can allow the generation of cell-specific and protein-specific reaction network models. Once constructed, a variety of routines are available in MATLAB for the deterministic and stochastic simulation of such networks. Sophisticated optimization methods are also available for the purpose of experimental data fitting. Complex post-simulation analysis can also be performed in this environment with the goal of generating experimentally testable hypotheses. Depending on the specific biological questions being addressed, such analysis is feasible both for the quantitative simulation of features regulating glycan expressions on the cell-surface and for the analysis of glycan heterogeneity on specific proteins. Enzyme definitions and rate constants for such pathway generation have to be curated from literature. Many examples of such glycosylation network analysis are available in literature and facilities are available in GNAT to curate these. Examples include, but are not limited to, mathematical models of N-linked glycosylation branching in Chinese Hamster Ovary cells [15], extended N-linked glycosylation models that include glycan maturation, extension and terminal capping by sialic acid or fucose [13], comparative simulations of glycosylation in different reactor configurations like plug-flow and continuous stirred tank reactors [25], and models of glycan heterogeneity on monoclonal antibodies including sugar-nucleotide biosynthesis processes [26]. Finally, while the examples provided in this work relate to O- and N-linked glycosylation, the general framework may also be extended to studies of glycolipid and glycosaminoglycan biosynthesis.

The range of problems that can be studied using the computer framework described in the current manuscript is large, and a few examples are provided below for the sake of completion. First, the cellular glycome depends on the sub-cellular location of individual enzymes, specific enzymatic activity, substrate availability and sugar-nucleotides levels. The effect of each of these features can be studied in GNAT by either specifying the compartment where specific enzymes are located, varying their rate constants or by altering the concentration of reactants and sugar-nucleotides during simulations. Second, beyond considering glycomics based datasets, the computational framework can also be extended to capture genomics, transcriptomics and metabolomics based data that describe changes in cellular gene expression and sugar-nucleotide synthesis. Such advancements can be implemented using the MATLAB scripting language and the resulting findings could be relevant to metabolic disorders and also cancer. Third, the *Enz* definition in GNAT may be modified in order to incorporate peptide backbone effects on glycosyltransferase activity and specificity.

In conclusion, the streamlined enzyme definitions enable the rapid construction of glycosylation reaction networks *in silico*, using both conventional biochemical and high-throughput MS experimental data. The implementation of these algorithms in GNAT provides an open-source computational framework for the generation of experimentally testable hypotheses.
